# Effects of *MAT1-2* Spore Ratios on Fruiting Body Formation and Degeneration in the Heterothallic Fungus *Cordyceps militaris*

**DOI:** 10.3390/jof9100971

**Published:** 2023-09-27

**Authors:** Tao Xuan Vu, Hanh-Dung Thai, Bich-Hang Thi Dinh, Huong Thi Nguyen, Huyen Thi Phuong Tran, Khanh-Linh Thi Bui, Tram Bao Tran, Hien Thanh Pham, Linh Thi Dam Mai, Diep Hong Le, Huy Quang Nguyen, Van-Tuan Tran

**Affiliations:** 1 National Key Laboratory of Enzyme and Protein Technology, University of Science, Vietnam National University, Hanoi (VNU), 334 Nguyen Trai, Thanh Xuan, Hanoi 100000, Vietnam; taovx.tsa@gmail.com (T.X.V.); hanhdungthai@gmail.com (H.-D.T.);; 2 Center for Experimental Biology, National Center for Technological Progress, Ministry of Science and Technology, C6 Thanh Xuan Bac, Thanh Xuan, Hanoi 100000, Vietnam; 3 Faculty of Biology, University of Science, Vietnam National University, Hanoi (VNU), 334 Nguyen Trai, Thanh Xuan, Hanoi 100000, Vietnam

**Keywords:** *Cordyceps militaris*, degeneration, fruiting body formation, heterokaryotic strains, heterothallic fungus, mating-type loci, sexual reproduction, spore ratios, successive culturing generations

## Abstract

The medicinal mushroom *Cordyceps militaris* is widely exploited in traditional medicine and nutraceuticals in Asian countries. However, fruiting body production in *C. militaris* is facing degeneration through cultivation batches, and the molecular mechanism of this phenomenon remains unclear. This study showed that fruiting body formation in three different *C. militaris* strains, namely G12, B12, and HQ1, severely declined after three successive culturing generations using the spore isolation method. PCR analyses revealed that these strains exist as heterokaryons and possess both the mating-type loci, *MAT1-1* and *MAT1-2*. Further, monokaryotic isolates carrying *MAT1-1* or *MAT1-2* were successfully separated from the fruiting bodies of all three heterokaryotic strains. A spore combination of the *MAT1-1* monokaryotic isolate and the *MAT1-2* monokaryotic isolate promoted fruiting body formation, while the single monokaryotic isolates could not do that themselves. Notably, we found that changes in ratios of the *MAT1-2* spores strongly influenced fruiting body formation in these strains. When the ratios of the *MAT1-2* spores increased to more than 15 times compared to the *MAT1-1* spores, the fruiting body formation decreased sharply. In contrast, when *MAT1-1* spores were increased proportionally, fruiting body formation was only slightly reduced. Our study also proposes a new solution to mitigate the degeneration in the heterokaryotic *C. militaris* strains caused by successive culturing generations.

## 1. Introduction

*Cordyceps* species are entomopathogenic ascomycete fungi. The *Cordyceps* genus comprises over 400 species that mainly parasitize in arthropods. Many species of this fungal genus are able to produce abundant bioactive substances with beneficial effects on human health [1,2,3,4]. Among *Cordyceps* species, *Cordyceps militaris* is cultivated artificially at a large scale for fruiting bodies. Fruiting bodies of *C. militaris* contain valuable bioactive ingredients such as cordycepin, adenosine, pentostatin, polysaccharides, and digestive enzymes. Therefore, sexual fruiting bodies of this fungus have been widely consumed as edible and medicinal ingredients in Asian countries, including Vietnam [5,6,7,8].

*C. militaris* is a heterothallic fungus whose sexual reproduction to form fruiting bodies requires both opposite mating-type partners, *MAT1-1* and *MAT1-2*. A heterokaryotic strain (a dikaryon/diploid) harbors both *MAT1-1* and *MAT1-2* nuclei, and single-ascospore isolation can help to obtain haploidic *MAT1-1* and *MAT1-2* isolates (monokaryons/haploids) from the parental heterokaryotic strain [9,10]. The *MAT1-1* and *MAT1-2* loci contain transcription factor genes required for mating and sexual development. Structural analysis of these mating-type loci in *C. militaris* revealed that the *MAT1-1* locus contains the *MAT1-1-1* and *MAT1-1-2* genes, while the *MAT1-2* locus carries only the *MAT1-2-1* gene. The accurate functioning of this mating system results in the formation of complete fruiting bodies as perithecial stromata [9,11]. Deletion of *MAT1-1* or *MAT1-2* resulted in impairment of the fruiting body formation in *C. militaris* [9]. Strikingly, some single *MAT1-1* mating-type strains of *C. militaris*, *Cordyceps takaomontana*, and *Cordyceps cardinalis* can also form fruiting bodies, but these stromata are sterile, without perithecia or ascospores [11,12,13]. The molecular mechanism for this phenomenon is still not known yet.

Successive subculturing of mycelia or spores for several generations in filamentous fungi and mushrooms causes degeneration [12,13,14,15,16,17,18]. Strain degeneration in *C. militaris* is characterized by the reduction in fruiting body formation, mycelial growth rate, pigmentation, and production of bioactive metabolites [19,20]. Degeneration of fruiting body formation can occur after two to ten consecutive subculturing generations via mycelial plug passages, depending on the tested strains [21,22,23]. However, the mechanism of degeneration in *C. militaris* is complex and remains unclear. It may involve environmental factors, genetic variations, and changes in gene expression [21,23,24]. In this study, we showed that the heterokaryotic *C. militaris* strains, including G12, B12, and HQ1, degenerated after three consecutive culturing generations by using the spore isolation method and effects of the *MAT1-2* mating-type spores on the degeneration of fruiting body formation in these strains.

## 2. Materials and Methods

### 2.1. Fungal Strains

Five *C. militaris* strains, namely G12, H8, M5, B12, and HQ1, were provided by the National Key Laboratory of Enzyme and Protein Technology, University of Science, Vietnam National University, Hanoi. These strains were preserved in 20% glycerol at 4 °C.

### 2.2. Media for Fungal Cultivation

The potato dextrose broth (PDB) medium comprised the infusion broth with 200 g peeled potato, 20 g glucose, and distilled water to 1000 mL. To this medium, 2% agar was added, and it was named potato dextrose agar (PDA).

Brown rice (BR) medium was used for the fruiting body formation of *C. militaris* strains. This medium, including 28 g brown rice and 60 mL of a nutrient solution, was added to a 600 mL round transparent plastic box for fungal inoculation. The nutrient solution comprised the infusion broth from 200 g peeled potato, 15 g glucose, 15 g sucrose, 1 g MgSO_4_, 1 g KH_2_PO_4_, 1 g peptone, 100 g finely ground silkworm pupae biomass, and distilled water to 1000 mL.

All media were autoclaved at 121 °C for 20 min before use.

### 2.3. Isolation of Ascospores from Fruiting Bodies

A single fruiting body of each heterokaryotic *C. militaris* strain was mounted on the inside lid of a Petri dish with sterile sticky tape as previously reported [25]. The Petri dish used for the fruiting body mounting contained the PDA medium supplemented with 100 mg/L chloramphenicol. The plate was incubated at 25 °C for 4 days under continuous illumination by fluorescent lamps with a 250–300 lux light intensity. Ascospores (sexual spores) from the fruiting body dropped on the surface of the PDA medium and formed colonies. Single colonies were separately transferred to new PDA plates and grown at 25 °C in the dark for 4 days. The plates were then exposed to continuous lighting for 2 days to promote conidia (asexual spores). Conidia were collected and washed with sterile distilled water after filtration through Miracloth (Calbiochem, Darmstadt, Germany) and by centrifugation at 6000 rpm (SIGMA 3K30, Sartorius AG, Göttingen, Germany) for 10 min at 4 °C. The concentration of the spore suspension was adjusted to 10^6^ spores/mL and stored at 4 °C for further experiments.

### 2.4. Total DNA Extraction

Fruiting body samples obtained from the artificial cultivation of five *C. militaris* strains (G12, H8, M5, B12, and HQ1) were chopped and used directly as the starting material for DNA extraction. Total DNA was also extracted from fungal mycelium prepared: one milliliter of the spore suspension (10^6^ spores/mL) was added to a 100 mL Erlenmeyer flask containing 50 mL of the PDB medium. The flask was incubated in an orbital shaker at 200 rpm and 25 °C for 3 days. The culture was filtered through Miracloth to collect fungal mycelial biomass.

Total DNA extraction was adapted from our previous work with slight adjustments [26]. Briefly, 200 mg of fungal biomass (fungal mycelium or fruiting bodies) was distributed to a 2 mL tube. The sample was well pounded with a clean metal rod or pulverized in liquid nitrogen. A volume of 600 µL of the extraction buffer (2.5% SDS, 200 mM Tris-HCl pH 8, 250 mM NaCl, 25 mM EDTA, 0.2% β-mercaptoethanol) was added to the tube. The tube was strongly vortexed and incubated at 60 °C for 30 min. Next, 300 µL of 3M sodium acetate (pH 5.2) was added, and the sample was mixed by inverting several times. The tube was centrifuged at 12,000 rpm (Mikro 185, Hettich, Tuttlingen, Germany) for 20 min at 4 °C. The supernatant phase was transferred to a new 1.5 mL tube. One equal volume of cold isopropanol was added to the tube to precipitate DNA. The tube was gently inverted several times and centrifuged at 12,000 rpm for 10 min at 4 °C. The obtained pellet was washed with 700 µL of 70% ethanol and centrifuged at 12,000 rpm for 5 min. The pellet was dried in the SpeedVac system (Thermo Scientific, Waltham, MA, USA) and dissolved in 30 µL of TE buffer (Tris-EDTA, pH 8). Then, 3 µL of RNase A (10 mg/mL) was added to the tube, which was incubated at 60 °C for 30 min to eliminate RNA. The DNA sample was quantified using NanoDrop (Thermo Scientific, Waltham, MA, USA) and stored at −20 °C for further experiments.

### 2.5. Identification of the Mating-Type Genes by PCR

Total DNA samples extracted from fungal materials of the *C. militaris* strains were used for PCR to detect the mating-type genes *MAT1-1-1* (for the *MAT1-1* locus) and *MAT1-2-1* (for the *MAT1-2* locus). PCR amplifications involved GoTaq^®^ Green Master Mix (Promega, Fitchburg, WI, USA) and two specific primer pairs, MAT1-1-1-F (5′-ATGGAACACAGATCGAGCGACAC-3′)/MAT1-1-1-R (5′-ATATACCTTCGCGATCATT GCCCAG-3′) and MAT1-2-1-F (5′-TGTTTTGTCGCGATGGTTCTGG-3′)/MAT1-2-1-R (5′-CCTCTGGAGGTTCTGCATTCCA-3′) [25]. The steps of the PCR procedure included 94 °C (6 min); 35 cycles of 94 °C (30 s), 58 °C (30 s), 72 °C (40 s); and 72 °C (10 min). PCR products were analyzed on agarose gels with electrophoresis.

### 2.6. Assays for the Fruiting Body Formation through Successive Culturing Generations

Five *C. militaris* strains were consecutively grown on the PDA medium for five generations. First, an original strain was cultivated on a PDA plate to collect new spores (1st generation). Next, the harvested spores were grown to harvest new spores (2nd generation). This cycle was repeated to have the next generations (3rd, 4th, 5th). All five generations of each *C. militaris* strain were used to evaluate the fruiting body formation ability. One milliliter of a spore suspension (10^6^ spores/mL) from each generation was inoculated into a flask containing the PDB medium supplemented with 0.1% peptone (HiMedia Laboratories, Maharashtra, India). The flask was kept in a shaking incubator at 200 rpm and 25 °C in the dark for 3 days. A volume of 3 mL of the culture was spread evenly onto the surface of the brown rice (BR) medium in a 600 mL round transparent plastic box (Viet Nhat Plastic, Hanoi, Vietnam). After the inoculation, the box was incubated at 25 °C in the dark for 7 days to promote the colonization of fungal mycelium to the entire surface of the substrate. The box was transferred to culture conditions of a lighting cycle of 12 h under the light/12 h in the dark, a light intensity of 500–700 lux, a temperature of 22 °C, and a humidity of 85–90%. The box was maintained under these conditions for 45 days to promote fruiting body formation.

### 2.7. Assays for Effects of Spore Ratios on Fruiting Body Formation

Monokaryotic isolates carrying *MAT1-1* or *MAT1-2* derived from the heterokaryotic *C. militaris* strains (G12, B12, and HQ1) were mixed in pairwise combinations to examine fruiting body formation. Spores from each *MAT1-1* isolate and each *MAT1-2* isolate were combined with different ratios: 1:1, 1:5, 1:10, 1:15, 1:20, 1:25, 1:30, 5:1, 10:1, 15:1, 20:1, 25:1, and 30:1. Each mixed spore ratio was added to a conical flask containing PDB medium and grown at 200 rpm and 25 °C for 3 days. Obtained cultures were inoculated to the BR medium to promote fruiting body formation as described above. Fruit bodies were harvested, and the parameters of height, weight, and number of fruit bodies from each culture box were documented for comparative analyses.

### 2.8. Maintaining Fruiting Body Formation in the Heterokaryotic Strains

The *MAT1-1* and *MAT1-2* monokaryotic isolates derived from the heterokaryotic *C. militaris* strains (G12, B12, and HQ1) were cultured separately on the PDA medium for five successive generations using the spore isolation method. For each generation, the *MAT1-1* isolate was mixed with the *MAT1-2* isolate at the 1:1 ratio and grown in the PDB medium supplemented with 0.1% peptone (HiMedia Laboratories, Maharashtra, India). The mixed culture was spread on the BR medium to promote fruiting body formation as described above. The effectiveness of fruiting body formation was evaluated through height, weight, and number of fruiting bodies per culture box.

### 2.9. Statistical Analysis

Data were expressed as means ± standard deviations and analyzed statistically using one-way analysis of variance (ANOVA). Analyses were performed with GraphPad Prism 8 (GraphPad Software, San Diego, CA, USA) using Tukey’s test. A statistical difference was considered when *p* < 0.05.

## 3. Results and Discussion

### 3.1. Heterokaryotic C. militaris Strains Carrying Both Mating-Type Loci Degenerate through Consecutive Culturing Generations

**Figure 1:**
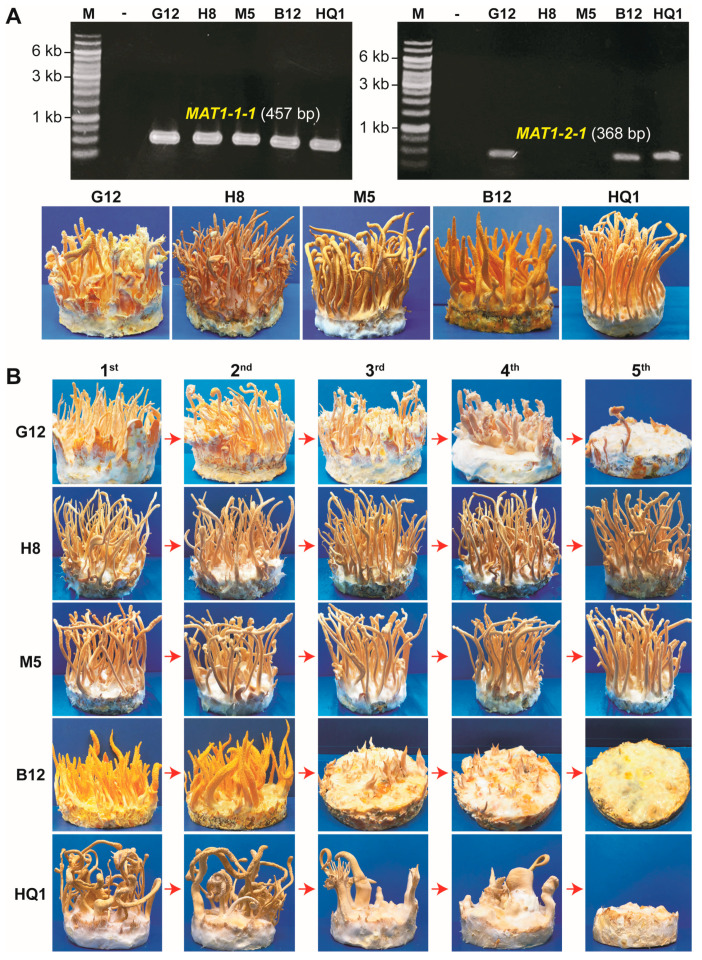
Fruiting body formation in different *C. militaris* strains. (**A**) The mating-type loci (*MAT1-1*, *MAT1-2*) in five tested *C. militaris* strains (G12, H8, M5, B12, and HQ1) were examined with PCR using the primer pairs specific for the *MAT1-1-1* and *MAT1-2-1* genes. PCR products were analyzed on 0.7% agarose gels. All five *C. militaris* strains were artificially cultivated to evaluate the fruiting body formation ability. (**B**) The *C. militaris* strains were cultured on the PDA medium for five successive generations (first to fifth) using the spore isolation method. All five generations were inoculated to the BR medium with the culture conditions of 22 °C, a humidity of 85–90%, and a lighting cycle of 12 h light/12 h dark for 45 days to promote fruiting body formation.

We first examined the mating-type loci (*MAT1-1* and *MAT1-2*) in all five tested *C. militaris* strains (G12, H8, M5, B12, and HQ1) with PCR using the primers specific for the *MAT1-1-1* and *MAT1-2-1* genes. Results showed that strains H8 and M5 carried only the *MAT1-1* mating-type locus, while G12, B12, and HQ1 were confirmed as heterokaryotic strains carrying both the mating-type loci, *MAT1-1* and *MAT1-2*, in their genomes. Artificial cultivation of these *C. militaris* strains on the BR medium revealed that all five strains could form normal fruiting bodies (Figure 1A). These strains were further evaluated for their ability to form fruiting bodies through five successive generations. Results indicated that all these strains could form normal fruiting bodies in the two first generations. However, only two monokaryotic strains (H8 and M5) carrying *MAT1-1* could maintain the ability to produce fruiting bodies from the third to the fifth generation. In contrast, the fruiting body formation in three heterokaryotic strains (G12, B12, and HQ1) was strongly decreased (Figure 1B).

Although sexual reproduction in heterothallic *Cordyceps* species usually requires both *MAT1-1* and *MAT1*-2 for fruiting body production [9,11,12,13], Zheng et al. (2011) also indicated that the sequenced *C. militaris* strain (Cm01) carrying only *MAT1-1* without an opposite mating-type partner in its genome is still able to form fruiting bodies (stromata). However, these fruiting bodies are incomplete due to the lack of perithecia (the flask-shaped fruiting structure) and ascospores [11]. This phenomenon was also discovered in some haploidic *MAT1-1* strains of *C. takaomontana* and *C. cardinalis* [12,13]. In our study, monokaryotic strains H8 and M5 harboring the *MAT1-1* mating-type locus were more stable for fruiting body formation than heterokaryotic strains G12, B12, and HQ1 through successive culturing generations (Figure 1). These strains appear to resemble the sequenced Cm01 strain. The monokaryotic strain Cm01 is culturally stable and commercialized in China [11].

In agreement with a previous study [23], our results also showed that the degeneration in all three heterokaryotic strains (G12, B12, and HQ1) of *C. militaris* began in the third generation and was more severe in the fourth generation. In the fifth generation, fruiting body formation in these strains was completely impaired (Figure 1B).

### 3.2. Both the Mating-Type Loci, MAT1-1 and MAT1-2, Are Required for Fruiting Body Formation in the Heterokaryotic C. militaris Strains

Using the fruiting body mounting method [25], we successfully isolated ascospores from the fruiting bodies of all three heterokaryotic *C. militaris* strains (G12, B12, and HQ1). Fungal colonies developed from ascospores were examined for the mating-type loci *MAT1-1* and *MAT1-2*. PCR results showed that some colonies carried only a single mating-type locus as *MAT1-1* or *MAT1-2*. However, we also found that many colonies still existed as heterokaryons carrying both *MAT1-1* and *MAT1-2*. Perhaps it is difficult to separate single ascospores from each other by using the fruiting body mounting method.

**Figure 2:**
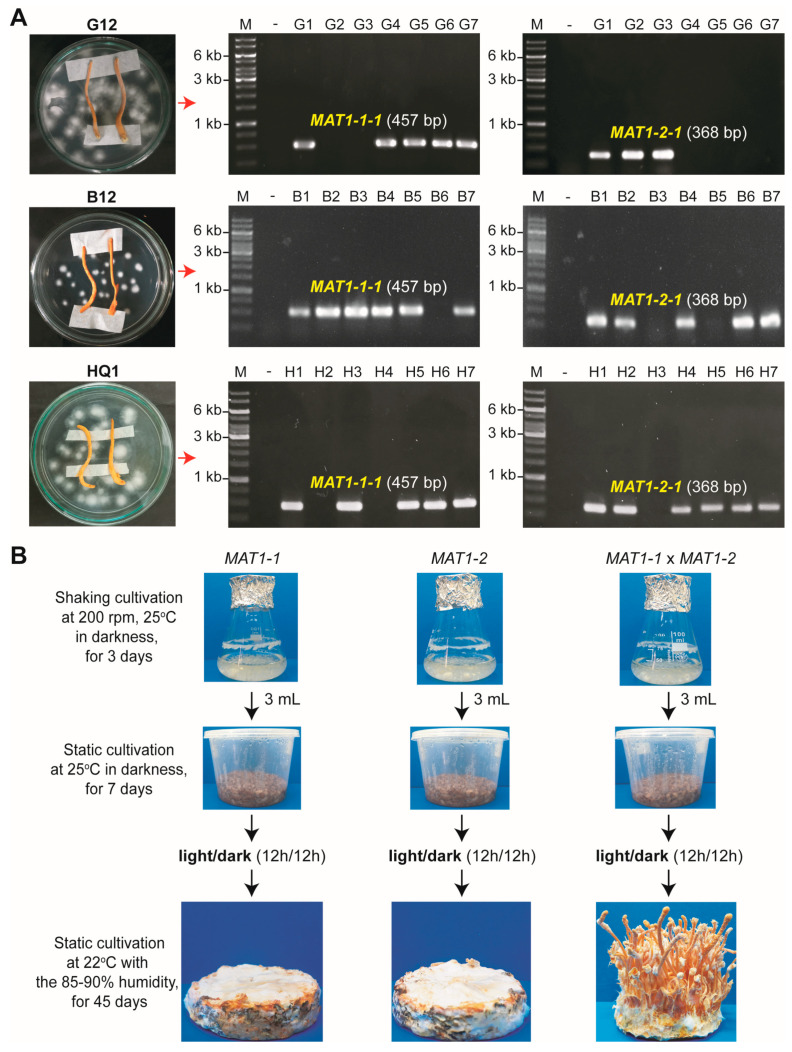
Examining opposite mating-type ascospores from the heterokaryotic strains for fruiting body formation. (**A**) Isolation of ascospores from three heterokaryotic strains, G12, B12, and HQ1. Fungal colonies derived from ascospores were confirmed for the mating-type loci (*MAT1-1* and *MAT1-2*) by PCR. (**B**) Monokaryotic isolates from a representative heterokaryotic strain (G12) were cultured as separated or combined to evaluate fruiting body formation. Fruiting body formation assays were conducted on the BR medium for 45 days at 22 °C, with a humidity of 85–90% and a lighting cycle of 12 h light/12 h dark.

We selected six colonies as monokaryotic (haploid) isolates derived from the heterokaryotic strains (G12, B12, and HQ1) for fruiting body formation assays. These monokaryotic isolates included G4 (*MAT1-1*) and G2 (*MAT1-2*) from strain G12, B5 (*MAT1-1*) and B6 (*MAT1-2*) from strain B12, and H3 (*MAT1-1*) and H2 (*MAT1-2*) from strain HQ1 (Figure 2A). Previously, Zheng et al. (2011) reported that the inoculation of *MAT1-1* or *MAT1-2* monokaryotic isolates obtained from the hybrid *C. militaris* Cm06 strain on caterpillar pupae resulted in the formation of sterile fruiting bodies lacking perithecia and ascospores [11]. However, no fruiting body could be formed in our study when the monokaryotic isolates derived from heterokaryotic strains G12, B12, and HQ1 were cultivated separately on the BR medium. Instead, a combination of a *MAT1-1* isolate and a *MAT1-2* isolate was required to induce the fruiting body formation on the BR medium (Figure 2B). Our results resemble the ones reported for the heterokaryotic *C. militaris* Cm09 strain [9].

In *MAT1-1* and *MAT1-2* mating-type loci structures, the most conserved mating-type genes are *MAT1-1-1* and *MAT1-2-1*, respectively. These gene sequences were also employed for phylogenetic analyses to identify species of Clavicipitaceae, including *Cordyceps* species [27]. Inactivation of *MAT1-1* or *MAT1-2* by deleting the respective genes resulted in the loss of fruiting body formation in *C. militaris* [9]. These results revealed the essential roles of both the mating-type loci in developing complete fruiting bodies in *C. militaris*. However, the effects of *MAT1-1* and *MAT1-2* proportions on fruiting body formation in *C. militaris* still need to be clarified.

### 3.3. Excessive Increase in MAT1-2 Spore Ratios Caused the Degeneration in the Heterokaryotic Strains

Sung et al. (2006) inspected the effect of different ratios of two mono-ascospore strains in pairwise combinations on fruiting body formation in *C. militaris*. The results indicated that fruiting bodies were similarly produced when two monokaryotic strains were combined at the ratios of 1:1, 1:2, 1:3, 1:4, 2:1, 3:1, and 4:1 [21]. Later, Zheng et al. (2011) also reported that the inoculation of the mixed spores of the *MAT1-1* and the *MAT1-2* monokaryotic isolates at the ratios of 1:1, 1:9, and 9:1 formed similarly sexual fruiting bodies (stromata) [11]. Based on these data, it seems that the combination of the opposite mating-type monokaryotic strains at different ratios is not necessary for stromata production.

**Figure 3:**
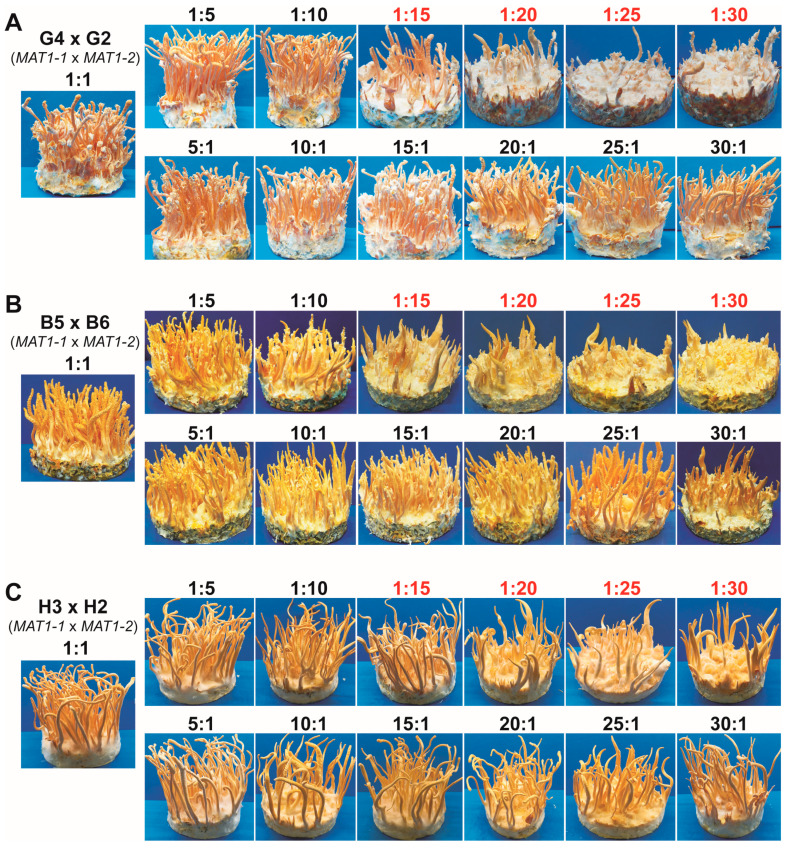
Effects of different mating-type spore ratios in combinations on fruiting body formation in three heterokaryotic *C. militaris* strains. (**A**) Combinations of monokaryotic isolates G4 (*MAT1-1*) and G2 (*MAT1-2*) derived from strain G12. (**B**) Combinations of monokaryotic isolates B5 (*MAT1-1*) and B6 (*MAT1-2*) derived from strain B12. (**C**) Combinations of monokaryotic isolates H3 (*MAT1-1*) and H2 (*MAT1-2*) derived from HQ1. Spore mixtures were inoculated on the BR medium for 45 days at 22 °C, with a humidity of 85–90% and a lighting cycle of 12 h light/12 h dark to promote the formation of fruiting bodies.

In agreement with the previous works, all three heterokaryotic strains in our study could form similar fruiting bodies when two opposite mating-type monokaryotic isolates were combined (*MAT1-1* × *MAT1-2*) at the spore ratios of 1:1, 1:5, 1:10, 5:1, 10:1, 15:1, 20:1, 25:1, and 30:1. However, it was astonishing that the formation of fruiting bodies strongly declined in the combinations at the ratios of 1:15, 1:20, 1:25, and 1:30. These results revealed that an excessive increase in the *MAT1-2* spores (over 15 times) compared to the *MAT1-1* spores led to the degeneration in the heterokaryotic strains of *C. militaris*. In contrast, an excessive increase in the *MAT1-1* spores (even 30 times) of the combination only caused a slight reduction in fruiting body formation (Figure 3, Supplementary Materials Figure S1).

Zheng et al. (2011) showed that the *MAT1-1*/*MAT1-2* heterokaryotic Cm06 strain produced normal stromata with perithecia and ascospores. PCR analysis of 30 single ascospore isolates derived from Cm06 revealed that 28 single ascospore isolates carried the *MAT1-1* mating type, and only 2 isolates contained the *MAT1-2* mating type [11]. This unequal distribution of the mating types corresponds to a 14:1 spore ratio. Both the *MAT1-1* and *MAT1-2* mating types are essential for fruiting body formation in the heterokaryotic strains [9,11]. However, a highly excessive number of *MAT1-2* spores can cause degeneration (Figure 3). A recent comparative analysis with quantitative real-time PCR also indicated that the expression of the *MAT1-2-1* gene in a degenerated *C. militaris* strain was significantly higher than in its parental strain [28]. Based on this information and our results, we suggest that the abundance of the *MAT1-1* monokaryotic spores in the mating combinations can support the stability of the fruiting body formation in the heterokaryotic *C. militaris* strains.

### 3.4. Preserving the Monokaryotic Isolates Separately before Mating Mitigates the Degeneration in the Heterokaryotic Strains Caused by Successive Culturing

**Figure 4:**
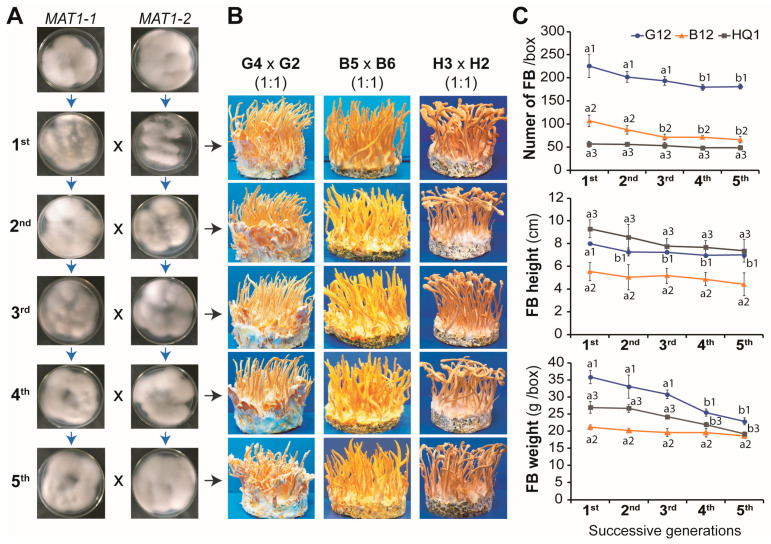
Maintaining the fruiting body formation ability of the heterokaryotic strains. (**A**) Monokaryotic isolates of the *MAT1-1* mating type and the *MAT1-2* mating type were preserved separately. These isolates were successively cultured for five generations. The opposite mating-type spores from each generation were mixed and cultivated on the BR medium for fruiting body formation. (**B**) Fruiting body formation in three heterokaryotic strains (G12, B12, and HQ1) after five successive culturing generations of the monokaryotic isolates and respective spore combinations by mating at a 1:1 ratio. (**C**) Quantification of fruiting bodies from the combinations. Three parameters, namely number of fruiting bodies (FBs), height of FBs, and weight of FBs, were documented. Experiments were conducted in triplicate, and data are presented as means ± standard deviations. Error bars represent the standard deviations, and different lowercase letters indicate significant differences (*p* < 0.05).

Degeneration in heterokaryotic *C. militaris* strains occurs after a few generations of successive subculturing, and the formation of fruiting bodies can even be lost entirely [21,22,23]. We cultured the *MAT1-1* and *MAT1-2* monokaryotic isolates for five consecutive generations using the spore isolation method. The monokaryotic isolates from each generation were then combined at a 1:1 spore ratio. Our results revealed that the formation of fruiting bodies in the hybrid strains was similarly maintained through all five generations (Figure 4). Previous studies reported that monokaryotic isolates derived from single ascospores of heterokaryotic *C. militaris* strains were more stable for fruiting body production [10,21]. Our work further showed that the *MAT1-1* and *MAT1-2* monokaryotic isolates should be individually preserved as compatible partners for mating to mitigate the degeneration of fruiting body formation during cultivation.

## 4. Conclusions

This study showed that the sexual (fertile) fruiting body formation in the *MAT1-1*/*MAT1-2* heterokaryotic strains of *C. militaris* quickly degenerated after three successive generations via the asexual spore (conidia) isolation. In contrast, the sterile fruiting body formation in the *MAT1-1* monokaryotic strains was more stable through consecutive culturing generations. We also discovered that an excessive increase in the *MAT1-2* spores of over 15 times compared with the *MAT1-1* spores led to the degeneration of fruiting body formation in all three examined heterokaryotic *C. militaris* strains (G12, B12, and HQ1). To mitigate the degeneration in the heterokaryotic strains through culturing generations, their monokaryotic isolates should be separately kept before being combined for fruiting body production.

## Data Availability

All data for this study are included in the manuscript.

## Supplementary Materials

The following supporting information can be downloaded at: https://www.mdpi.com/article/10.3390/jof9100971/s1, Figure S1: Quantification of fruiting body formation for mating-type spore combinations at different ratios.

## References

[1] Sung G.-H., Hywel-Jones N.L., Sung J.-M., Luangsa-ard J.J., Shrestha B., Spatafora J.W. (2007). Phylogenetic classification of *Cordyceps* and the clavicipitaceous fungi. Stud. Mycol..

[2] Ng T.B., Wang H.X. (2005). Pharmacological actions of *Cordyceps*, a prized folk medicine. J. Pharm. Pharmacol..

[3] Das G., Shin H.-S., Leyva-Gómez G., Prado-Audelo M.L.D., Cortes H., Singh Y.D., Panda M.K., Mishra A.P., Nigam M., Saklani S. (2021). *Cordyceps* spp.: A review on its immune-stimulatory and other biological potentials. Front. Pharmacol..

[4] Wang L., Yan H., Zeng B., Hu Z. (2022). Research progress on cordycepin synthesis and methods for enhancement of cordycepin production in *Cordyceps militaris*. Bioengineering.

[5] Phull A.-R., Ahmed M., Park H.-J. (2022). *Cordyceps militaris* as a bio functional food source: Pharmacological potential, anti-inflammatory actions and related molecular mechanisms. Microorganisms.

[6] Wang Y., Dong Q.-Y., Luo R., Fan Q., Duan D.-E., Dao V.-M., Wang Y.-B., Yu H. (2023). Molecular phylogeny and morphology reveal cryptic species in the *Cordyceps militaris* complex from Vietnam. J. Fungi.

[7] Shweta, Abdullah S., Komal, Kumar A. (2023). A brief review on the medicinal uses of *Cordyceps militaris*. Pharmacol. Res. Mod. Chin. Med..

[8] Krishna K.V., Ulhas R.S., Malaviya A. (2023). Bioactive compounds from *Cordyceps* and their therapeutic potential. Crit. Rev. Biotechnol..

[9] Lu Y., Xia Y., Luo F., Dong C., Wang C. (2016). Functional convergence and divergence of mating-type genes fulfilling in *Cordyceps militaris*. Fungal Genet. Biol..

[10] Shrestha B., Kim H.-K., Sung G.-H., Spatafora J.W., Sung J.-M. (2004). Bipolar heterothallism, a principal mating system of *Cordyceps militaris* in vitro. Biotechnol. Bioprocess Eng..

[11] Zheng P., Xia Y., Xiao G., Xiong C., Hu X., Zhang S., Zheng H., Huang Y., Zhou Y., Wang S. (2012). Genome sequence of the insect pathogenic fungus *Cordyceps militaris*, a valued traditional Chinese medicine. Genome Biol..

[12] Yokoyama E., Yamagishi K., Hara A. (2005). Heterothallism in *Cordyceps takaomontana*. FEMS Microbiol. Lett..

[13] Sung G.-H., Shrestha B., Han S.-K., Kim S.-Y., Sung J.-M. (2010). Heterothallic type of mating system for *Cordyceps cardinalis*. Mycobiology.

[14] Danner C., Mach R.L., Mach-Aigner A.R. (2023). The phenomenon of strain degeneration in biotechnologically relevant fungi. Appl. Microbiol. Biotechnol..

[15] Chen X., Zhang Z., Liu X., Cui B., Miao W., Cheng W., Zhao F. (2019). Characteristics analysis reveals the progress of *Volvariella volvacea* mycelium subculture degeneration. Front. Microbiol..

[16] Pérez G., Lopez-Moya F., Chuina E., Ibañez-Vea M., Garde E., López-Llorca L.V., Pisabarro A.G., Ramírez L. (2021). Strain Degeneration in *Pleurotus ostreatus*: A genotype dependent oxidative stress process which triggers oxidative stress, cellular detoxifying and cell wall reshaping genes. J. Fungi.

[17] Zhao F., Liu X., Chen C., Cheng Z., Wang W., Yun J. (2022). Successive mycelial subculturing decreased lignocellulase activity and increased ROS accumulation in *Volvariella volvacea*. Front. Microbiol..

[18] Du X.-H., Zhao Q., Xia E.-H., Gao L.-Z., Richard F., Yang Z.L. (2017). Mixed-reproductive strategies, competitive mating-type distribution and life cycle of fourteen black morel species. Sci. Rep..

[19] Shrestha B., Zhang W., Zhang Y., Liu X. (2012). The medicinal fungus *Cordyceps militaris*: Research and development. Mycol. Prog..

[20] Sun S.-J., Deng C.-H., Zhang L.-Y., Hu K.-H. (2017). Molecular analysis and biochemical characteristics of degenerated strains of *Cordyceps militaris*. Arch. Microbiol..

[21] Sung J.-M., Park Y.-J., Lee J.-O., Han S.-K., Lee W.-H., Choi S.-K., Shrestha B. (2006). Effect of preservation periods and subcultures on fruiting body formation of *Cordyceps militaris* in vitro. Mycobiology.

[22] Xiong C., Xia Y., Zheng P., Wang C. (2012). Increasing oxidative stress tolerance and subculturing stability of *Cordyceps militaris* by overexpression of a glutathione peroxidase gene. Appl. Microbiol. Biotechnol..

[23] Yin J., Xin X., Weng Y., Gui Z. (2017). Transcriptome-wide analysis reveals the progress of *Cordyceps militaris* subculture degeneration. PLoS ONE.

[24] Lou H., Lin J., Guo L., Wang X., Tian S., Liu C., Zhao Y., Zhao R. (2019). Advances in research on *Cordyceps militaris* degeneration. Appl. Microbiol. Biotechnol..

[25] Kang N., Lee H.-H., Park I., Seo Y.-S. (2017). Development of high cordycepin-producing *Cordyceps militaris* strains. Mycobiology.

[26] Tran V.T., Do T.B.X.L., Nguyen T.K., Vu X.T., Dao B.N., Nguyen H.H. (2017). A simple, efficient and universal method for the extraction of genomic DNA from bacteria, yeasts, molds and microalgae suitable for PCR-based applications. Vietnam. J. Sci. Technol. Eng..

[27] Yokoyama E., Arakawa M., Yamagishi K., Hara A. (2006). Phylogenetic and structural analyses of the mating-type loci in Clavicipitaceae. FEMS Microbiol. Lett..

[28] Wellham P.A.D., Hafeez A., Gregori A., Brock M., Kim D.-H., Chandler D., de Moor C.H. (2021). Culture degeneration reduces sex-related gene expression, alters metabolite production and reduces insect pathogenic response in *Cordyceps militaris*. Microorganisms.

