# Supplementary material for: Effects of MAT1-2 Spore Ratios on Fruiting Body Formation and Degeneration in the Heterothallic Fungus Cordyceps militaris
Source: J Fungi (Basel). 2023 Sep 27;9(10):971. doi: 10.3390/jof9100971 (PMC10607669; doi:10.3390/jof9100971)
Supplement: Supplementary file 1 [file jof-09-00971-s001.zip › jof-2602975-supplementary.pdf]

Journal of Fungi

Effects of *MAT1-2* Spore Ratios on Fruiting Body Formation and Degeneration in the Heterothallic Fungus *Cordyceps militaris*

Tao Xuan Vu [1,2], Hanh-Dung Thai [1], Bich-Hang Thi Dinh [1], Huong Thi Nguyen [1], Huyen Thi Phuong Tran [1], Khanh-Linh Thi Bui [1], Tram Bao Tran [2], Hien Thanh Pham [3], Linh Thi Dam Mai [3], Diep Hong Le [3], Huy Quang Nguyen [1,3] and Van-Tuan Tran [1,3,*]

Supplementary Materials

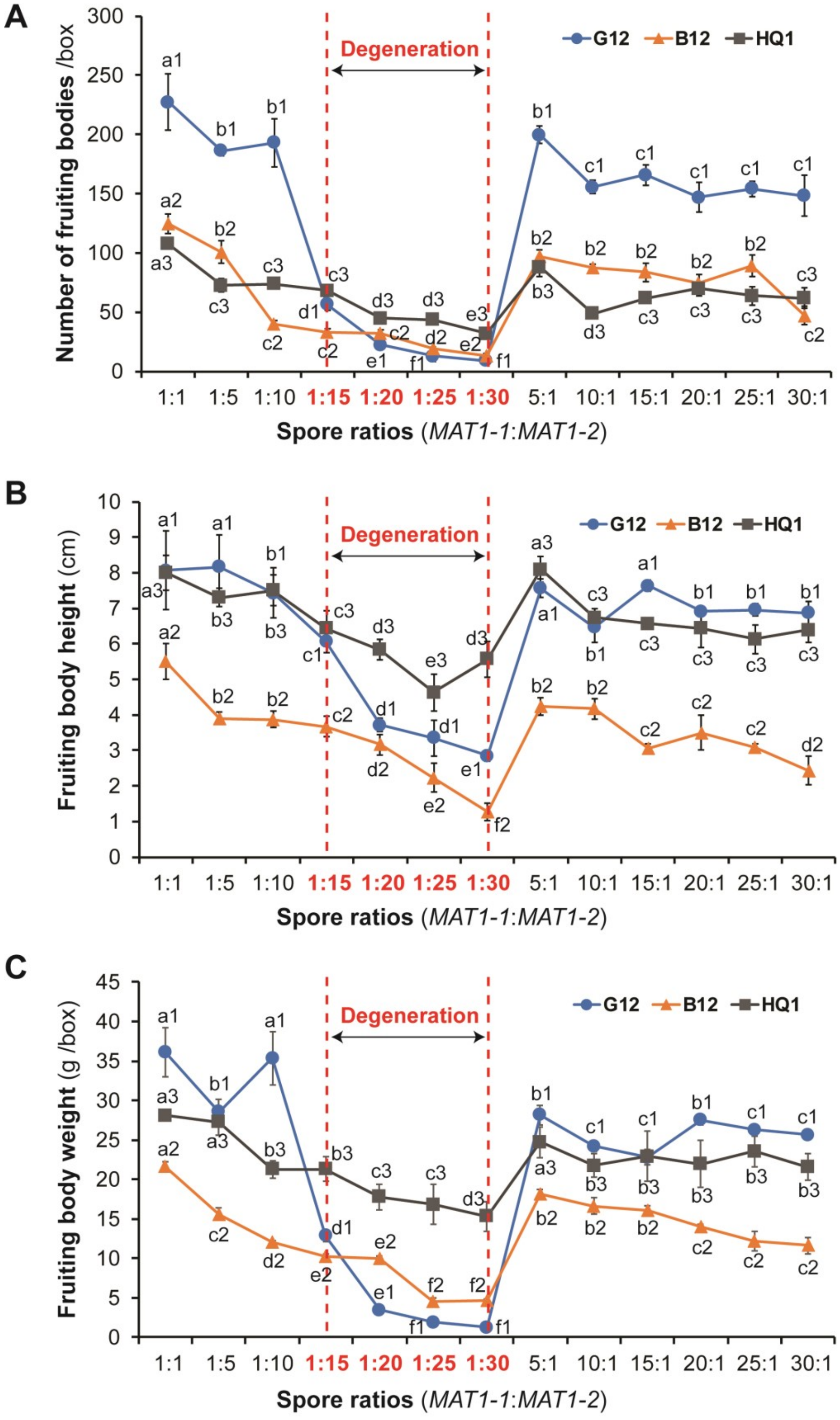

Figure S1. Quantification of fruiting body formation for mating-type spore combinations at different ratios. (**A**) Number of fruiting bodies. (**B**) Height of fruiting bodies. (**C**) Weight of fruiting bodies. Experiments were conducted in triplicate, and data are presented as means ± standard deviations. Error bars represent the standard deviations, and different lowercase letters indicate significant differences ($p < 0.05$).
